# Comparison of the Skin Microbiota in the Periocular Region between Patients with Inflammatory Skin Diseases and Healthy Participants: A Preliminary Study

**DOI:** 10.3390/life14091091

**Published:** 2024-08-30

**Authors:** Iva Ferček, Petar Ozretić, Arjana Tambić-Andrašević, Slave Trajanoski, Diana Ćesić, Marko Jelić, Goran Geber, Orjena Žaja, Josipa Paić, Liborija Lugović-Mihić, Rok Čivljak

**Affiliations:** 1 Department of Ophthalmology, Zabok General Hospital and Croatian Veterans’ Hospital, 49210 Zabok, Croatia; 2 Laboratory for Hereditary Cancer, Division of Molecular Medicine, Ruđer Bošković Institute, 10000 Zagreb, Croatia; pozretic@irb.hr; 3 School of Dental Medicine, University of Zagreb, 10000 Zagreb, Croatia; arjana.tambic@bfm.hr (A.T.-A.); gorangeber@kbcsm.hr (G.G.); ozaja@sfzg.hr (O.Ž.); liborija@sfzg.hr (L.L.-M.); 4 Department of Clinical Microbiology, University Hospital for Infectious Diseases, 10000 Zagreb, Croatia; mjelic@bfm.hr; 5 Core Facility Computational Bioanalytics, Center for Medical Research, Medical University Graz, 8010 Graz, Austria; slave.trajanoski@medunigraz.at; 6 Department of Dermatology and Venereology, Medikol Clinic, 10000 Zagreb, Croatia; dianavrancic@gmail.com; 7 Department of Otorhinolaryngology and Head and Neck Surgery, Sestre Milosrdnice University Hospital Centre, 10000 Zagreb, Croatia; 8 Department of Pediatrics, Sestre Milosrdnice University Hospital Centre, 10000 Zagreb, Croatia; 9 Department of Ophthalmology and Optometry, Šibenik General Hospital, 22000 Šibenik, Croatia; josipa.paic@gmail.com; 10 Department of Dermatology and Venereology, Sestre Milosrdnice University Hospital Centre, 10000 Zagreb, Croatia; 11 Department for Respiratory Infections, University Hospital for Infectious Diseases “Dr. Fran Mihaljević”, 10000 Zagreb, Croatia; rok.civljak@bfm.hr; 12 School of Medicine, University of Zagreb, 10000 Zagreb, Croatia

**Keywords:** periocular dermatitis, atopic, seborrheic, rosacea, contact, skin microbiota, Gram-positive anaerobic cocci, *Corynebacterium*

## Abstract

(1) Background: Periocular or periorbital dermatitis is a common term for all inflammatory skin diseases affecting the area of skin around the eyes. The clear etiopathogenesis of periocular dermatitis is still not fully understood. Advances in molecular techniques for studying microorganisms living in and on our bodies have highlighted the microbiome as a possible contributor to disease, as well as a promising diagnostic marker and target for innovative treatments. The aim of this study was to compare the composition and diversity of the skin microbiota in the periocular region between healthy individuals and individuals affected by the specific entity of periocular dermatitis. (2) Methods: A total of 35 patients with periocular dermatitis and 39 healthy controls were enrolled in the study. After a skin swab from the periocular region was taken from all participants, DNA extraction and 16S rRNA gene amplicon sequencing using Illumina NovaSeq technology were performed. (3) Results: *Staphylococcus* and *Corynebacterium* were the most abundant bacterial genera in the microbiota of healthy skin. Analysis of alpha diversity revealed a statistically significant change (*p* < 0.05) in biodiversity based on the Faith’s PD index between patients and healthy individuals. We did not observe changes in beta diversity. The linear discriminant analysis effect size (LEfSe) revealed that *Rothia*, *Corynebacterium*, *Bartonella,* and *Paracoccus* were enriched in patients, and *Anaerococcus*, *Bacteroides*, *Porphyromonas*, and *Enhydrobacter* were enriched in healthy controls. (4) Conclusions: According to the results obtained, we assume that the observed changes in the bacterial microbiota on the skin, particularly Gram-positive anaerobic cocci and skin commensals of the genus *Corynebacterium*, could be one of the factors in the pathogenesis of the investigated inflammatory diseases. The identified differences in the microbiota between healthy individuals and patients with periocular dermatitis should be further investigated.

## 1. Introduction

Chronic inflammatory skin diseases represent the most common group of skin conditions. Periocular or periorbital dermatitis (PD) is a term used to describe all inflammatory skin diseases affecting the area around the eyes and/or the eyelid skin. The clinical presentation of PD includes redness of the affected skin, sometimes accompanied by mild edema, as well as skin scaling in chronic forms of the disease. In addition to the aesthetically unacceptable redness, patients often report itching, tenderness, and a feeling of tightness of the affected skin [1].

The differential diagnosis of periocular dermatitis includes non-infectious diseases such as contact dermatitis, atopic dermatitis, seborrheic dermatitis, rosacea, psoriasis [2,3,4,5,6,7,8], as well as certain infectious diseases such as erysipelas, impetigo, syphilis, herpes zoster/shingles, human immunodeficiency virus infection, and other localized and/or systemic bacterial, viral, and fungal infections [1]. Additionally, many connective tissue diseases and autoimmune diseases can present with periocular skin lesions, such as discoid lupus erythematosus and dermatomyositis. Furthermore, the skin around the eyes is often involved in rejuvenation procedures (e.g., dermal filler injections and laser applications), which may lead to hypersensitivity reactions, infections, foreign body sensation, delayed erythema, and hypo- or hyperpigmentation [2,9]. The eyelids, being integral to the eye’s protective system, are frequently affected by inflammatory conditions due to their unique structure. The skin around the eyes is particularly prone to allergic reactions because it is exceptionally thin (0.55 mm) compared to other facial areas (2 mm), making it more susceptible to allergen penetration [10]. The unprotected position of the eyelids contributes to their specific exposure to environmental factors, including frequent contact with allergens applied by hands; eyelids are also a common site for the application of various eye care products and medications.

The development of PD, as well as other inflammatory skin conditions, is associated with several factors, including impairment of the skin’s protective barrier, triggering of the body’s innate immune response, and alterations in the skin microbiome. Stress is increasingly recognized as a possible trigger of inflammation, either directly through the peripheral nervous system or indirectly through the endocrine and immune systems [11]. Research into the etiopathogenesis of inflammatory skin diseases is particularly focused on investigating the role of the skin microbiome in the development of disorders of skin defense systems, which normally contribute to homeostasis and the preservation of normal skin structure and health. The term “microbiome” comprises the totality of microorganisms (microbiota), their genomes, and the environmental factors in a particular environment [12]. Changes in the composition and/or functionality of the human skin microbiota are also believed to cause immune dysregulation and, consequently, an inflammatory response, potentially playing a significant role in the clinical manifestations of inflammatory skin diseases [12]. Most research in this area has focused on atopic dermatitis, while studies in seborrheic dermatitis, psoriasis, acne, rosacea, and pruritus are less common [13,14,15,16,17,18].

Based on previous research findings [19], it is now understood that our skin harbors four dominant bacterial phyla whose relative abundance depends on body site: Actinobacteria, Firmicutes, Proteobacteria, and Bacteroidota. More than 200 bacterial genera have been identified, with *Corynebacterium*, *Cutibacterium*, and *Staphylococcus* being the most common. In terms of bacterial density, the skin ranks second only to the gut [20]. Through various chemical processes, the skin provides nutrients and a favorable environment for its commensal bacteria.

Data on the composition of the skin microbiome in the periocular region are scarce, with only a few studies examining the microbiome of healthy eyelid skin. The aim of our research was to investigate the composition of the microbiota of healthy skin in the periocular region and compare it with the microbiota of skin lesions in patients with specific entity of PD: patients with atopic dermatitis, seborrheic dermatitis, rosacea, and contact dermatitis. To our knowledge, this is the first study investigating the microbiota in inflammatory diseases of the periocular region. Understanding the microbiota composition could contribute to elucidating the etiopathogenesis of inflammatory skin diseases, streamline the diagnostic process, and potentially reduce treatment costs.

## 2. Materials and Methods

### 2.1. Study Design and Sample Collection

This observational case-control study was conducted at the University Hospital for Infectious Diseases “Dr. Fran Mihaljević” in Zagreb and Department of Dermatovenerology of the Clinical Hospital Center Sestre Milosrdnice in collaboration with the Ruđer Bošković Institute. The research was conducted in accordance with the Helsinki Declaration and was approved by the Ethics Committee of the University Hospital for Infectious Diseases “Dr. Fran Mihaljević” (Approval No 01-1327-4-2020, 26 June 2020) and Clinical Hospital Center Sestre Milosrdnice (Approval No 251-29-11-20-01-6, 13 February 2020). All participants provided informed consent before being included in the study.

The study included a total of 74 participants, out of which 35 were individuals diagnosed with PD, and 39 were healthy controls, including 52 women and 22 men. Inclusion criteria for affected participants were a clinical diagnosis of PD, characterized by periocular skin lesions not attributable to specific infectious entities, and age above 18 years. Patients were further classified into entity-specific groups: atopic dermatitis, seborrheic dermatitis, rosacea, and contact dermatitis. The control group consisted of healthy participants, either volunteers or individuals accompanying patients, aged above 18 years, matched for sex and age with the patients. Exclusion criteria for all participants included the use of systemic antibiotics, antifungals, antivirals, or antiparasitic drugs, corticosteroids, biological therapies, methotrexate, and immunosuppressive agents within one month prior to sample collection; application of topical antibiotics, corticosteroids, and calcineurin inhibitors to the periocular skin within seven days before sample collection; use of antimicrobial soaps and creams and swimming in chlorinated pools 24 h before sample collection; bathing and showering (with anything other than water) and applying creams, lotions, and similar preparations within 12 h before sample collection; intake of commercial probiotics; pregnancy, and breastfeeding.

For the analysis of bacterial microbiota, skin swabs were collected from the upper eyelid area of all participants. The microbiota of visible skin lesions on the upper eyelid was analyzed in patients with PD, while the microbiota of the upper eyelid skin without clinically visible skin lesions (intact skin) was analyzed in healthy participants. Sterile swabs with a synthetic tip Puritan HydraFlock (Puritan Medical Products, Guilford, ME, USA) with a breakable tip were used, which were immersed in sterile 0.15 M NaCl solution after opening and then pressed against the eyelid skin. The swab was taken by rubbing the skin for at least 30 s, rotating the swab across the entire eyelid skin to ensure an even distribution of the sample. Afterward, the tip of the swab was broken off and placed in a Zymo DNA/RNA Shield Lysis Tube (Zymo Research, Irvine, CA, USA), then immediately stored at −80 degrees until DNA extraction. To ensure consistency, all swab collection was performed by the same person. A blank sample, or blind probe, was obtained by immersing a swab into a tube containing sterile 0.15 M NaCl solution. The swab was then placed into a Zymo DNA/RNA Shield Lysis Tube and processed in the same manner as the other samples collected from the subjects.

### 2.2. DNA Extraction, PCR Amplification, and 16S rRNA Gene Sequencing

DNA extraction from skin swab samples was performed using the ZymoBIOMICS DNA Miniprep Kit (Zymo Research) following the manufacturer’s instructions.

The initial step involved homogenizing the samples in Zymo DNA/RNA Shield Lysis Tubes using the FastPrep FP120 Cell Disrupter device (Thermo Electron Corporation, Milford, MA, USA) twice for 45 s at a speed of 6.5 m/s, with a 5 min interval during which the lysis tubes were kept on ice. After sample homogenization, 250 μL of the supernatant was centrifuged and passed through DNA-binding column filters. This process was repeated several times with the addition of DNA wash buffer. Finally, microbial DNA was eluted once in 50 µL ZymoBIOMICS DNase/RNase-Free Water (Zymo Research). The DNA was quantified using a Qubit 4 Fluorometer (Thermo Fisher Scientific, Waltham, MA, USA) with the Qubit 1× dsDNA High Sensitivity (HS) Assay Kit Q33230 (Thermo Fisher Scientific).

Twenty microliters (20 µL) of DNA from the isolated samples was sent for 16S rRNA gene sequencing to Novogene (Novogene Co., Cambridge Science Park, Cambridge, UK). The DNA was diluted to 1 ng/μL using sterile water, depending on the concentration. The V3-V4 regions of the 16S rRNA gene were amplified using primers 341F (5′-CCTAYGGGRBGCASCAG-3′) and 806R (5′-GGACTACNNGGGTATCTAAT-3′) specific to these variable regions.

During the PCR reaction, 15 μL of Phusion^®^ High-Fidelity PCR Master Mix (New England Biolabs, Ipswich, MA, USA), 2 μM of primers, and 10 ng of DNA isolated from the samples were used. The thermal cycling consisted of an initial denaturation at 98 °C for 1 min, followed by 30 cycles of denaturation at 98 °C for 10 s, annealing at 50 °C for 30 s, and extension at 72 °C for 30 s, followed by a final extension at 72 °C for 5 min. An equal volume of buffer containing the fluorescent dye “SYBR green” was mixed with the PCR products, and electrophoresis was performed on a 2% agarose gel for detection. The PCR product mixture was then purified using the Qiagen Gel Extraction kit (Qiagen, Hilden, Germany). For library preparation, the TruSeq DNA PCR-Sample Preparation Kit (Illumina, San Diego, CA, USA) was used according to the manufacturer’s instructions. The library quality was assessed using a Qubit 2.0 fluorometer (Thermo Fisher Scientific) and Bioanalyzer 2100 (Agilent Technologies, Santa Clara, CA, USA). Finally, the library was sequenced on an Illumina NovaSeq platform (Illumina) to generate clusters and amplicons of 250-base-pair paired-end reads.

### 2.3. Bioinformatics and Statistical Analysis

Initial paired-end raw sequencing reads were checked for quality control and filtered with Cutadapt version 3.3 [21] and were then merged using the flash software version 1.2.11 [22] followed by tags filtration with Fastp version 0.23.1 [23] and chimera removal with the VSEARCH algorithm version 2.16.0 [24] against the SILVA rRNA database [25]. Resulting sequences were further processed using the UPARSE software version 7.0.1001 [26] to generate Operational Taxonomic Unit (OTU) clusters. Sequences with 97% similarity were assigned to the same OTU cluster. Taxonomic annotation of the representative sequences for each OTU cluster was performed with QIIME (Quantitative Insights Into Microbial Ecology) version 1.9.1 [27] against the SILVA 16s rRNA database [25]. To investigate the phylogenetic relationship between different OTUs and differences between dominant taxonomic groups in different samples (groups), multiple sequence alignment was conducted using the MUSCLE software version 3.8.1551 [28].

Alpha diversity was used to analyze the complexity of species diversity in a sample, using indices Faith’s phylogenetic diversity (PD), Shannon entropy, observed features, and Pielou’s evenness, calculated with QIIME v 1.9.1 and displayed with R software version 4.0.3 (Foundation for Statistical Computing, Vienna, Austria). Beta diversity was used to evaluate differences in species complexity between samples. Beta diversity on weighted Unifrac was calculated by QIIME software version 1.9.1. Cluster analysis was preceded by principal component analysis (PCA), which was applied to reduce the dimension of the original variables using the ade4 package and ggplot2 package in R software version 4.0.3. Principal coordinate analysis (PCoA) was performed to obtain principal coordinates and visualize from complex, multidimensional data and was displayed by ade4 package and ggplot2 package in R software version 4.0.3.

Differences in demographic characteristics were assessed using Mann–Whitney test (for age) and Fisher’s exact test (for sex) using MedCalc v22.030 (MedCalc Software Ltd., Ostend, Belgium). Two-tailed *p* values less than <0.05 with Bonferroni adjustment for subgroup comparisons were considered statistically significant.

LefSe analysis was conducted to investigate the statistically significant variances in the relative abundance of bacteria between groups. LefSe first identifies features that statistically differ between biological communities and then evaluates whether these differences are consistent with expected biological behavior through additional statistical tests, for example, whether a bacterial group is more abundant in all groups under investigation or only in one. It involves conducting non-parametric Kruskal–Wallis and Wilcoxon rank-sum tests, and finally linear discriminant analysis effect size (LDA) to assess the effect size of each differentially abundant feature [29]. LDA values greater than 2.0 and two-tailed *p* values less than 0.05 were considered statistically significant.

## 3. Results

The study enrolled participants aged 18 and older diagnosed with PD, while healthy individuals aged 18 and older without visible skin lesions were included as the control group. A total of 74 participants were enrolled, comprising 35 (47%) patients and 39 (53%) controls, including 52 (70%) women and 22 (30%) men. In the PD group, there were four subgroups of participants: atopic dermatitis (*n* = 12/34%), seborrheic dermatitis (*n* = 7/20%), rosacea (*n* = 10/29%), and contact dermatitis *(n* = 6/17%). A total of 74 skin swabs were collected, and after sample processing, an analysis of the skin bacterial microbiota was conducted. Demographic data are shown in Table 1. We observed no significant differences in sex and age between the whole PD cohort and healthy controls (*p* = 0.2199 and *p* = 0.4549, respectively). There were also no significant differences in sex between controls and each of the PD subgroup (*p* > 0.05). Although patients with atopic dermatitis appeared significantly younger than the controls (*p* = 0.0228), this difference was not statistically significant after Bonferroni adjustment (*p* > 0.0125). However, despite the potential for bias due to the relatively small sample size, the sex distribution in our PD cohort is consistent with the known fact that women are more frequently affected by these diseases [3,4,6,8].

### 3.1. Composition of Bacterial Skin Microbiota in PD Patients and Healthy Controls

**Figure 1:**
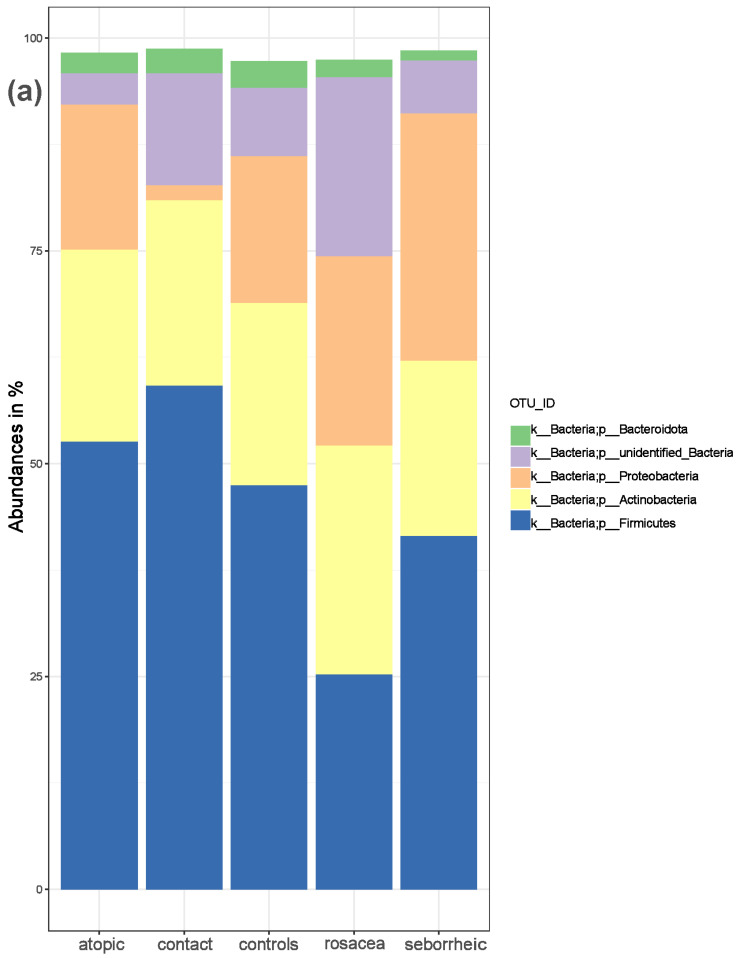

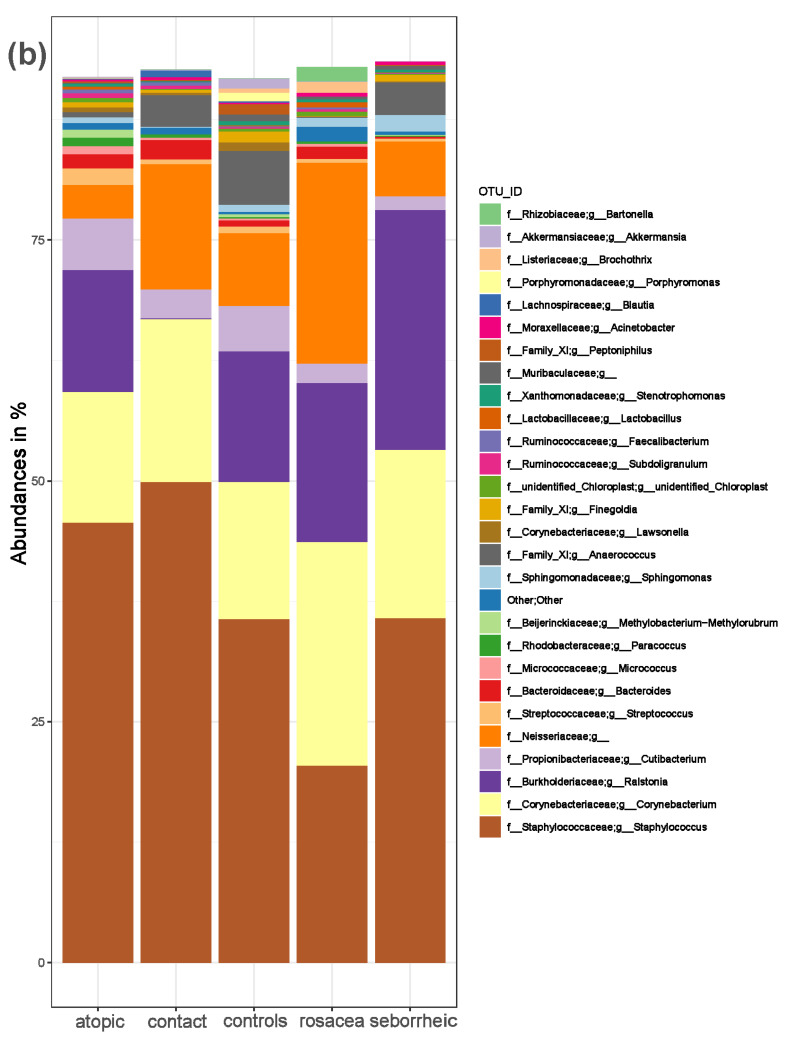
The composition of the skin microbiota in PD patients and healthy controls. (**a**) Relative abundance at the phylum and (**b**) at the genus level for healthy controls and patients with atopic dermatitis, contact dermatitis, rosacea, and seborrheic dermatitis.

Following quality assessment, skin samples from 74 subjects yielded a total of 7,731,103 effective reads, averaging 104,474.4 reads per sample. We identified 57 phyla, 127 classes, 283 orders, 441 families, 856 genera, and 598 species. We investigated the bacterial populations and their relative abundance in the healthy controls and disease subgroups across various taxonomic levels. The top 10 most abundant bacterial groups are shown in Figure 1 at the phylum and genus taxonomic levels. A threshold of 25 was used for the sum of prevalence across all groups at the phylum level, and a threshold of 1 was used for the genus level.

We identified Firmicutes (median = 47.4%) as the most abundant phylum in healthy controls, followed by Actinobacteria (median = 21.5%), Proteobacteria (median = 17.3%), and Bacteroidota (median = 3.2%). In patients with atopic dermatitis, Firmicutes (median = 52.6%) was the most abundant phylum, followed by Actinobacteria (median = 22.6%), Proteobacteria (median = 17.1%), and Bacteroidota (median = 2.4%). In patients with seborrheic dermatitis, the most abundant phyla were Firmicutes (median = 41.5%), Proteobacteria (median = 29.0%), Actinobacteria (median = 20.6%), and Bacteroidota (median = 1.2%). In patients with rosacea, Actinobacteria (median = 26.9%) and Firmicutes (median = 25.2%) were the most abundant phyla, followed by Proteobacteria (median = 22.3%), and Bacteroidota (median = 2.1%). In patients with contact dermatitis, Firmicutes (median = 59.1%) was the most abundant phylum, followed by Actinobacteria (median = 21.8%), Bacteroidota (median = 2.9%), and Proteobacteria (median = 1.8%). The most abundant genus was *Staphylococcus* in all participants, except in patients with rosacea, where *Corynebacterium* was predominant.

**Figure 2:**
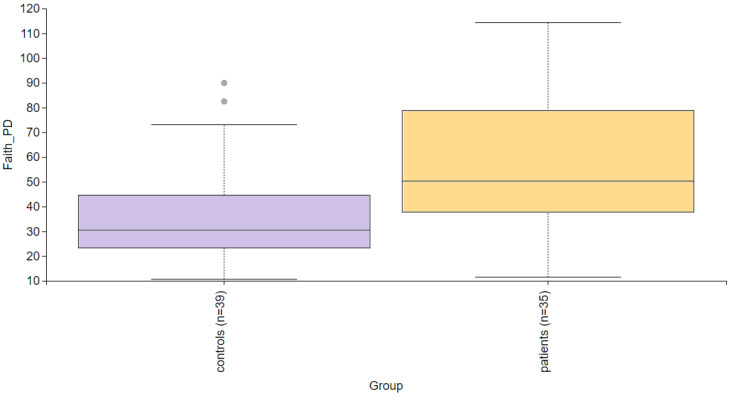
Alpha diversity comparison between the healthy controls and PD patients. Faith’s PD index revealed a statistically significant difference (*p* = 0.004).

**Figure 3:**
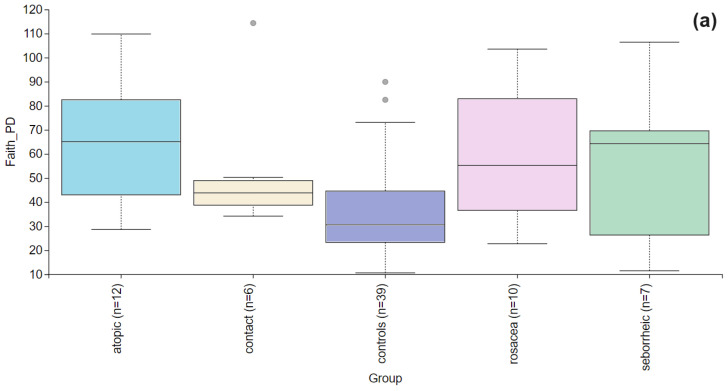

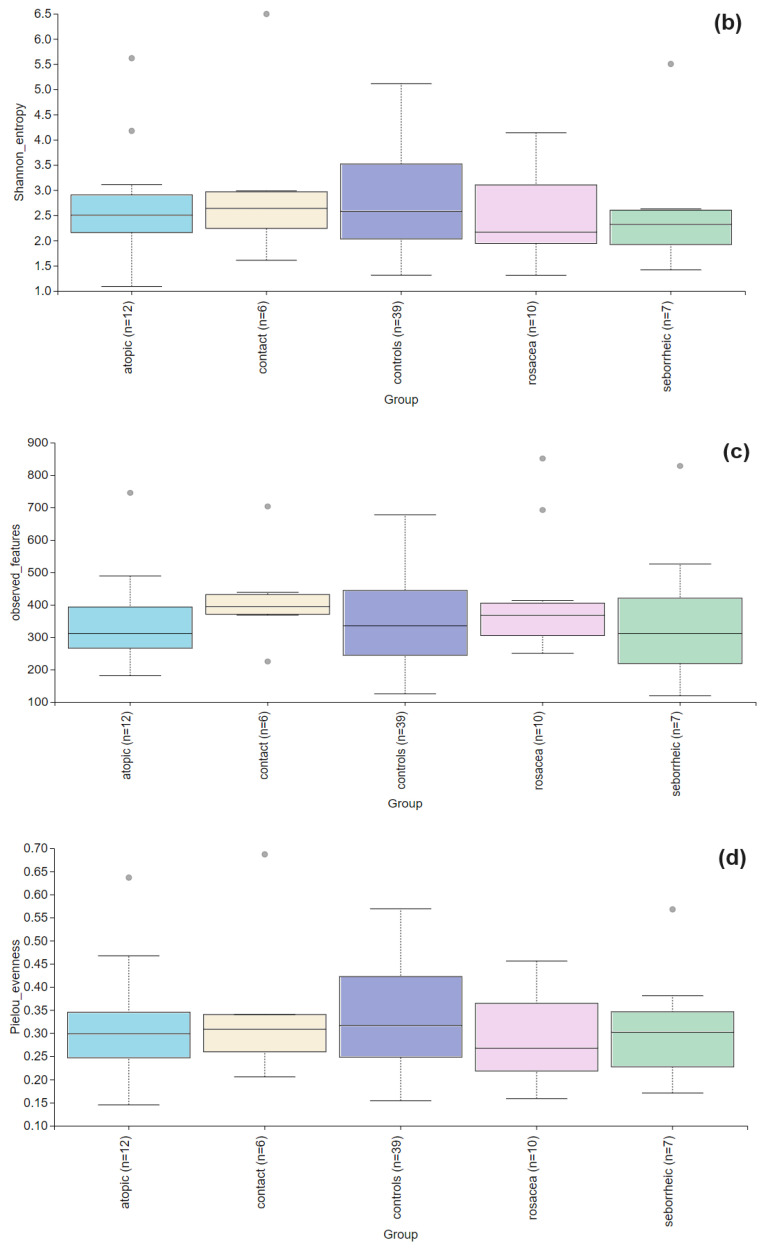
Alpha diversity comparison between the healthy controls and PD patients with atopic dermatitis, contact dermatitis, rosacea, and seborrheic dermatitis. (**a**) The Faith’s PD index showed a statistically significant difference in atopic dermatitis (*p* < 0.001) and rosacea (*p* = 0.013), while in contact dermatitis (*p* = 0.066) and seborrheic dermatitis (*p* = 0.278) there were no significant differences. (**b**) Shannon entropy index showed no significant differences between the healthy controls and PD patients with atopic dermatitis (*p* = 0.756), contact dermatitis (*p* = 0.947), rosacea (*p* = 0.472), and seborrheic dermatitis (*p*= 0.436). (**c**) Observed features index showed no significant differences between the healthy controls and PD patients with atopic dermatitis (*p* = 0.929), contact dermatitis (*p* = 0.256), rosacea (*p* = 0.315), and seborrheic dermatitis (*p* = 0.680). (**d**) Pielou evenness was similar across healthy controls and PD subgroups with no significant differences observed for atopic dermatitis (*p* = 0.641), contact dermatitis (*p* = 0.949), rosacea (*p* = 0.224), seborrheic dermatitis (*p* = 0.491).

Alpha diversity is used to analyze the structure of a community or sample in terms of its richness (the number of taxonomic groups) and evenness (the distribution of these groups) or both. Faith’s PD was significantly higher (*p* = 0.004) in the PD group compared to the control group (Figure 2). To further evaluate alpha diversity in the subgroups, we used Faith’s PD, Shannon entropy, observed features, and Pielou evenness indices, as shown in Figure 3. Faith’s PD was significantly higher in the atopic dermatitis (*p* < 0.001) and rosacea (*p* = 0.013) subgroups compared to the control group. Shannon entropy, observed features, and Pielou evenness indices did not show statistically significant differences but indicated a trend towards reduced biodiversity in patients with atopic dermatitis, seborrheic dermatitis, and rosacea. In contrast, patients with contact dermatitis exhibited a subtle trend towards increased biodiversity.

**Figure 4:**
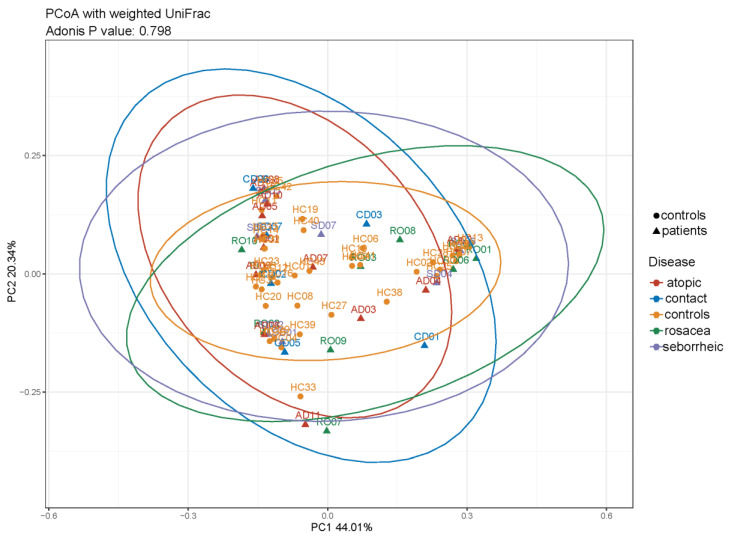
Beta diversity in healthy controls and PD subgroups based on principal coordinate analysis (PCoA) of weighted UniFrac distances (ADONIS, *p* = 0.796) showed overlap among all groups.

Beta diversity was used to evaluate differences in species complexity among samples. We examined the beta diversity of microbiota in PD patients and controls using principal coordinate analysis of weighted UniFrac distances. We did not observe clustering of samples among PD subgroups and controls, as shown in Figure 4.

### 3.2. The Statistical Analysis of the Most Abundant Taxonomic Groups

**Figure 5:**
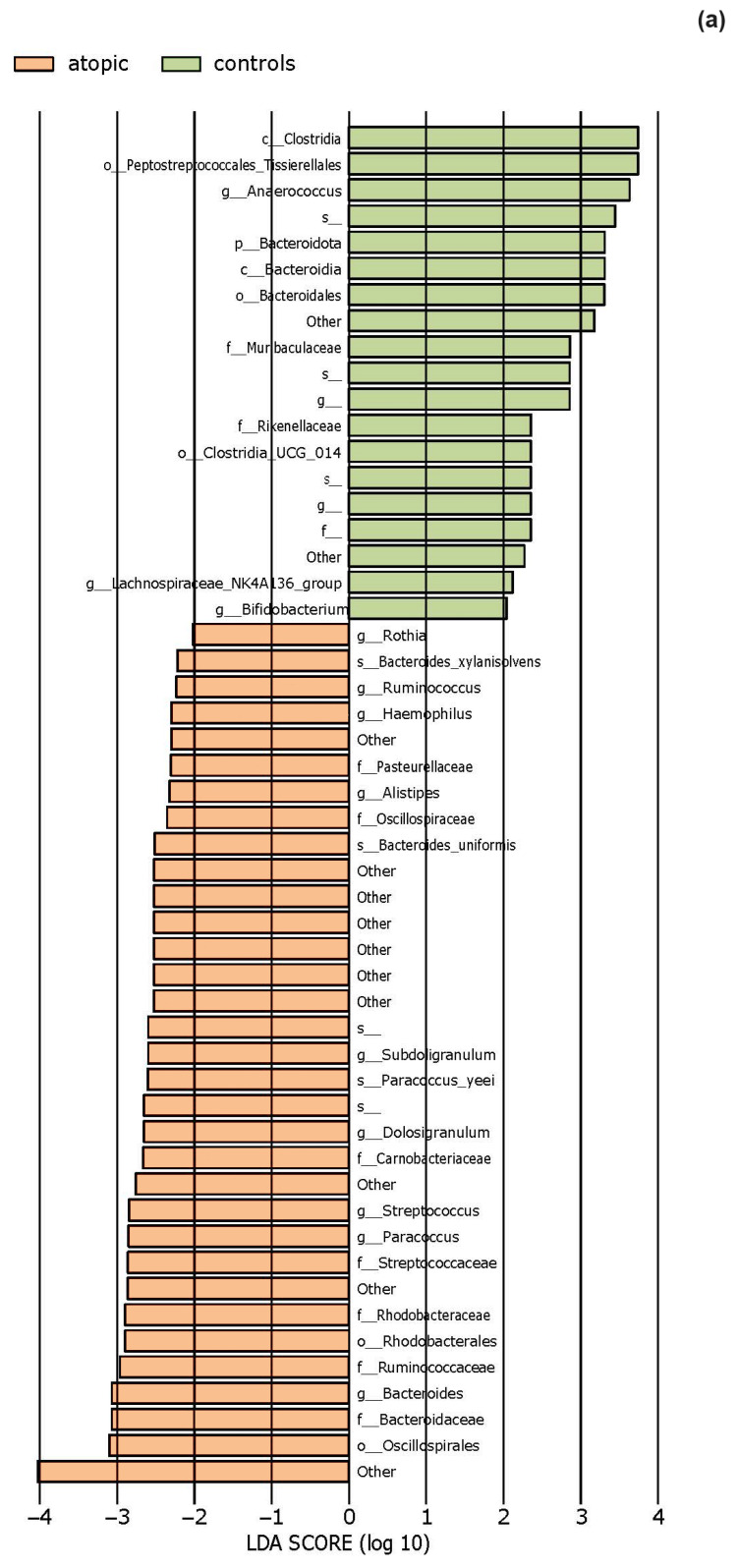

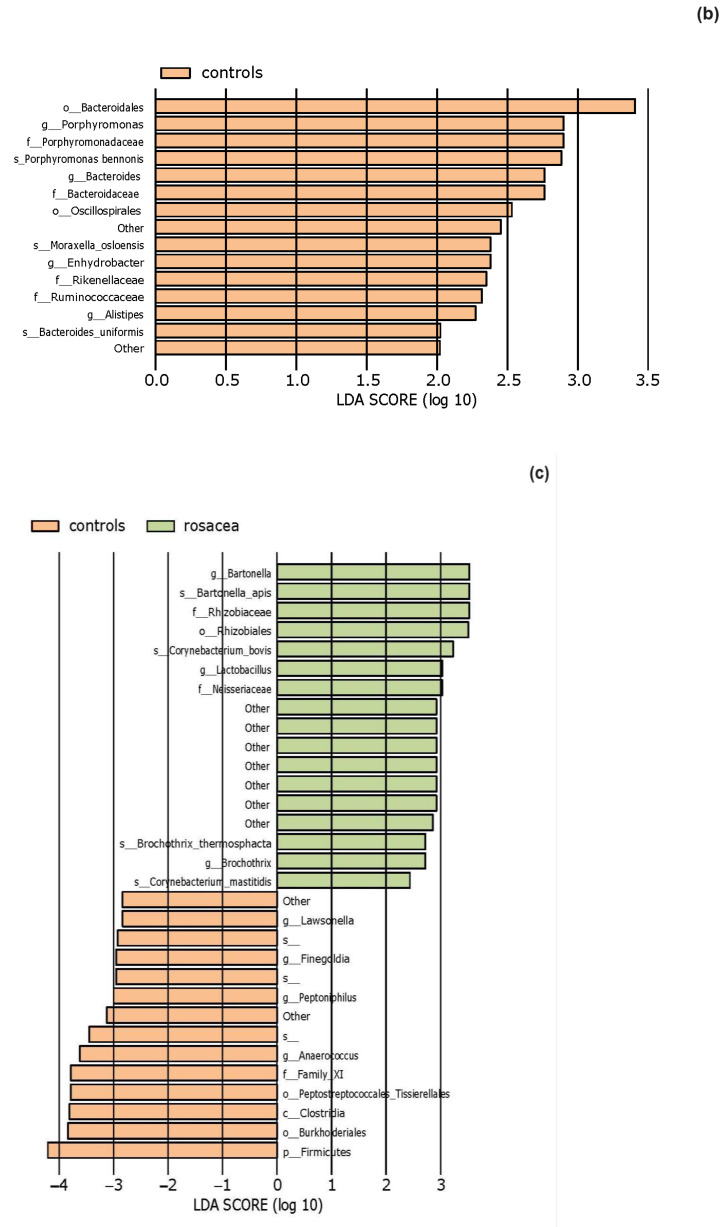

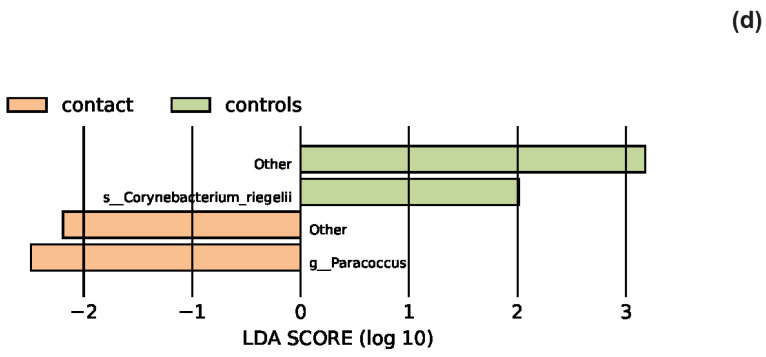
The LEfSe analysis highlighted the potential bacterial biomarkers. (**a**) In patients with atopic dermatitis, bacteria from various genera, including *Rothia,* were significantly more abundant, whereas those from genera *Anaerococcus* and *Bifidobacterium* were significantly decreased compared to controls. (**b**) In patients with seborrheic dermatitis, there was a significant decrease in the abundance of the genera *Bacteroides*, *Porphyromonas*, *Enhydrobacter*, and *Alistipes.* (**c**) In patients with rosacea, there was a significant increase in the abundance of the genus *Bartonella*, as well as *Corynebacterium bovis* and *C. mastitidis*, along with a decrease in the abundance of the genera *Anaerococcus* and *Finegoldia*. (**d**) In patients with contact dermatitis, the genus *Paracoccus* was significantly more abundant, while *C. bovis* was less abundant compared to controls.

The effect size of linear discriminant analysis (LDA) was used to identify key bacterial groups, with LDA scores greater than 2 being considered significant, as shown in Figure 5. The LEfSe analysis revealed that certain bacteria of the skin microbiota were significantly more or less abundant in the examined groups compared to healthy controls across various taxonomic levels. These bacteria could potentially serve as biomarkers for the investigated diseases.

## 4. Discussion

Chronic inflammatory skin diseases are the most prevalent type of skin conditions, affecting up to 25% of the population [30]. It is believed that, alongside changes in the skin barrier and immune system activation, numerous microorganisms residing on the skin and within organs could potentially play a role in the disease pathogenesis. Through this research, we identified the composition of the bacterial microbiota in healthy skin and in skin affected by inflammatory skin diseases, as well as differences in their composition. For a more detailed analysis of microbiota changes in the group of patients with PD, based on clinical presentation, as well as relevant laboratory and allergy tests, patients were divided in four disease subgroups: atopic dermatitis, seborrheic dermatitis, rosacea, and contact dermatitis. However, in clinical practice, the skin lesions of different diseases resemble each other and often several entities are present at the same time, such as atopic and contact dermatitis, which sometimes makes it difficult to establish an accurate diagnosis [31]. 

Our research results revealed that, in healthy individuals, the most abundant genus on the eyelid skin was *Staphylococcus,* belonging to the phylum Firmicutes, followed by the genus *Corynebacterium* (Proteobacteria). Previous studies on the microbiota of eyelid skin are limited and have not demonstrated differences in composition from the skin in other seborrheic areas [9,19]. Cavuoto et al. [9] demonstrated that the most abundant phylum on eyelid skin was Actinobacteria, followed by Firmicutes, Proteobacteria, and Bacteroidota. Suzuki et al. [32] found differences in the composition of skin microbiota between younger and older participants. In individuals under the age of 35, and the predominant genus was *Cutibacterium*, followed by *Staphylococcus*, while in those over the age of 65, the predominant genus was *Corynebacterium* and members of the *Neisseriaceae* family. Research on the microbiota of healthy skin across various facial locations revealed that the lips and eyelids, or periorificial areas, have a distinct microbiota composition compared to other facial locations, as well as greater biodiversity [33]. Our findings, in partial agreement with previous studies, demonstrated that the microbiota of eyelid skin is unique and differs in composition from other seborrheic areas. The studies mentioned sampled different skin locations: periocular skin [9], skin 3 mm below the lower eyelid margin [32], and the upper eyelid skin [33].

Our results, based on the Faith’s PD alpha diversity index, revealed differences in biodiversity within samples of healthy individuals and those with PD. Using three additional indices, we observed a trend toward reduced biodiversity in patients with atopic dermatitis, seborrheic dermatitis, and rosacea. This finding is consistent with the larger study conducted by Edslev et al. [34]. Meanwhile, patients with contact dermatitis showed a subtle trend toward increased biodiversity. Higher Faith’s PD index values observed in patients with PD indicate the presence of phylogenetically distant bacteria in their samples. This, along with the decrease in Shannon index, may suggest a low abundance of these diverse bacteria. 

We found no clear separation between PD patients and healthy controls based on the beta analysis. These results are consistent with the study by Bjerre et al. [35], which demonstrated differences in the composition of the bacterial microbiota in patients with atopic dermatitis on the hands, folds, and neck, while the microbiota on the feet and periocular and perioral areas was similar to that of healthy skin microbiota. Tao et al. [36] investigated the composition of bacterial microbiota in individuals with seborrheic dermatitis specifically focusing on facial lesions and found no significant change in beta diversity compared to healthy controls. Consistent with previous research [37,38,39,40], which found that patients with rosacea cluster together with their healthy controls, we did not observe changes in beta diversity in patients with rosacea.

### 4.1. Characteristics of the Skin Microbiota in Patients with Atopic Dermatitis

Based on 16S gene amplicon sequencing, we observed that on the periocular skin lesions in patients with atopic dermatitis, the most abundant phylum was Firmicutes, followed by Actinobacteria and Proteobacteria, while the most abundant genera were *Staphylococcus* and *Corynebacterium*. Previous studies [41,42] have also identified Firmicutes as the most abundant phylum and *Staphylococcus* as the most abundant genus in patients with moderate atopic dermatitis. LefSe revealed a statistically significant decrease in the abundance of the order Peptostreptococcales-Tissierellales as well as the genera *Anaerococcus* and *Bifidobacterium*. The observed reduction in commensal anaerobes is consistent with previous research that identified a depletion of the genera *Finegoldia*, *Anaerococcus*, and *Peptoniphilus* in patients with filaggrin protein mutations [43,44]. Using next-generation sequencing (NGS), these Gram-positive anaerobic cocci (GPAK) were identified as components of healthy skin and mucosa microbiota, utilizing protein degradation products for their metabolism [45].

Experimental results from Van der Krieken et al. [46] demonstrated that GPAK can induce a rapid innate immune response through 1. mononuclear cell-activated keratinocytes in circulating blood that directly regulate antimicrobial peptide (AMP) expression and pro-inflammatory mediators and 2. activation of the aryl hydrocarbon receptor (AhR). AhR is a ligand-dependent transcription factor expressed in numerous cells, including skin. Its precise role in the pathogenesis of inflammatory reactions is not yet fully understood, but AhR can initiate several signaling pathways involved in maintaining the skin barrier, regulating the terminal differentiation of CD4+ Th17 and Th22 cells, and the expression of IL-17 and IL-22 [47]. In conditions of Th-2 environment and altered bacterial microbiota composition found in patients with atopic dermatitis, there is a reduction in physiological AhR ligands. GPAK can activate the AhR signaling pathway in keratinocytes, potentially leading to increased antimicrobial activity and protective effects against *Staphylococcus aureus* colonization. During differentiation, filaggrin breaks down into a mixture of amino acids, including arginine and histidine, which serve as natural moisturizing factors, maintaining skin pH balance, and can serve as a nutrient source for bacteria using histidine as their carbon source, such as Gram-positive cocci [46]. In individuals with filaggrin gene mutations, levels of natural moisturizing factors within the *stratum corneum* are reduced [48]. Additionally, LefSe revealed that the genus *Rothia* was significantly more abundant in patients with atopic dermatitis compared to healthy controls, consistent with some previous research [49,50]. Bacteria from the genus *Rothia* are Gram-positive, non-motile, non-spore-forming, aerobic, or facultative anaerobic encapsulated bacteria belonging to the Actinobacteria phylum. They are considered opportunistic pathogens, especially in immunocompromised patients, and are associated with endocarditis, pneumonia, peritonitis, and septicemia [51]. This increase suggests that damage to the epidermal barrier may create conditions for the spread of potentially pathogenic bacteria at the expense of others whose abundance consequently decreases. Conversely, the decrease in the relative abundance of GPAK identified in our samples supports the notion that reduced GPAK levels compromise protective defense mechanisms against pathogenic bacteria in patients with atopic dermatitis.

### 4.2. Characteristics of the Skin Microbiota in Patients with Seborrheic Dermatitis

In patients with seborrheic dermatitis, we found that the most abundant phylum on lesional skin was Firmicutes, followed by Proteobacteria and most abundant genus was *Staphylococcus*, consistent with previously conducted studies [52,53,54,55].

Previous studies on the role of the microbiota in the pathogenesis of seborrheic dermatitis, using 16S rRNA sequencing methods [54,56,57], have focused on changes in the abundance of the bacterial genera *Cutibacterium* and *Staphylococcus*. These studies analyzed samples taken from the scalp. It was observed that, compared to healthy scalp, areas affected by seborrheic dermatitis have a reduced abundance of the genus *Cutibacterium* and increased abundance of the genus *Staphylococcus* [54,56,57]. This suggests that the balance between *Cutibacterium* and *Staphylococcus* may potentially contribute to the development of the disease [54]. However, our results demonstrated that the abundance of *Staphylococcus* was nearly equal between controls and patients. We observed a higher abundance of the genus *Cutibacterium* in healthy individuals compared to patients, but this depletion in patients was not statistically significant, in accordance with recent studies [36,52,53,58]. Additionally, research demonstrating that the reduction of *Staphylococcus* species using topical AMP (omiganan) does not lead to an improvement in clinical symptoms and signs of the disease [59] suggests that *Staphylococcus*, and perhaps bacterial dysbiosis, are not key factors in the pathogenesis of seborrheic dermatitis, as might be the case in patients with atopic dermatitis or rosacea. On the other hand, the already established treatment with ketoconazole led to an improvement in skin barrier function and a reduction in *Malassezia* yeast, again highlighting the imbalance of fungal communities and the disruption of the skin barrier in the development of this skin condition [59]. The role of *Cutibacterium* could be significant in patients with seborrheic dermatitis in the context of their complex interaction not only with other bacterial species but also with *Malassezia* yeast, which secrete lipases and contribute to changes in skin pH and disruption of the epidermal barrier.

According to LefSe analysis, we found a significant increase in the abundance of bacteria from the *Ruminococcaceae* family and the genera *Bacteroides*, *Porphyromonas*, *Enhydrobacter*, and *Alistipes* in healthy individuals compared to those with seborrheic dermatitis. Bacteria from the genera *Porphyromonas*, and *Enhydrobacter* are part of the healthy skin microbiome and rarely cause infections. The enrichment of the *Bacteroides* genus in healthy individuals is consistent with the findings of Lin et al. [53]. The effect of those bacteria on skin homeostasis is not yet well researched, unlike their role in promoting dysbiosis of the gut microbiome. The most abundant bacteria in the gut microbiome are Gram-negative and Gram-positive bacteria from the Bacteroidota and Firmicutes phyla, including the genera *Bacteroides*, *Bifidobacterium*, *Fusobacterium*, and *Ruminococcus*. These bacteria secrete short-chain fatty acids (SCFAs), which are believed to have anti-inflammatory properties [60]. SCFAs, produced by gut bacteria, reach distant organs through peripheral circulation, and bind to G protein-coupled receptors expressed on blood cells and skin cells, triggering signaling pathways involved in regulating the inflammatory response. They induce regulatory T cells that reduce the production of pro-inflammatory mediators such as TNF-α, IL-6, and IL-12 while increasing the production of anti-inflammatory mediators such as IL-10 [61,62]. Intestinal dysbiosis resulting from depletion of SCFA-producing bacteria is associated with the pathogenesis of inflammatory skin diseases such as atopic dermatitis, psoriasis, acne, chronic spontaneous urticaria, and food allergy [62,63,64,65]. Besides gut bacteria, bacteria residing on the skin, such as *Cutibacterium acnes*, are also thought to secrete SCFAs. Although the mechanisms by which *Cutibacterium acnes* produces propionic acid have not yet been fully elucidated, it is believed that it may influence the efficacy of the skin barrier through the secretion of SCFAs, which affect lipid production in keratinocytes and alter their composition [66].

### 4.3. Characteristics of the Skin Microbiota in Patients with Rosacea

Analysis of bacterial microbiota composition in patients with rosacea revealed that the most abundant phyla in rosacea patients were Actinobacteria and Firmicutes. The most abundant genera identified were *Corynebacterium* and *Staphylococcus*. Our results partially coincide with previous studies. For instance, some research [37,38,39] reported Actinobacteria as the most abundant phylum, with the *Cutibacterium* being the most abundant genus, while according to other [67], the most abundant genera in untreated rosacea skin were *Staphylococcus*, *Cutibacterium*, *Pseudomonas*, and *Corynebacterium*. On the other hand, our results do not align with the earlier study by Zaidi et al. [40], which found that the most abundant phyla in rosacea patients were Firmicutes and Proteobacteria.

We observed an increased abundance of the genus *Corynebacterium* in our patients with rosacea, consistent with previous research [37]. Specifically, species such as *C.acterium bovis* and *C. mastitidis* showed significant increases according to LefSe. Reiner et al. [37] reported an increased abundance of *C. kroppenstedtii*, a bacterium that lives in endosymbiosis with *Demodex folliculorum* [68], a mite believed to play a significant role in the pathogenesis of rosacea. Endosymbiosis is a specific type of symbiosis in which one, usually microbial, partner lives inside its host, benefiting both members of the symbiosis. *Demodex* is sometimes present on the skin of healthy individuals but is more commonly found in those with rosacea [69]. Research suggests that *Demodex* induces immunotolerance via dendritic cells, allowing it to persist on healthy skin. *Demodex* expresses the Thomsen-nouveau (Tn Ag) antigen and interacts with dendritic cells via their macrophage galactose-type lectin (MGL), which then migrate to lymph nodes and interact with naive T cells, inducing immunotolerance by presenting peptide antigens with major histocompatibility complex (MHC) class II on naive T-cell receptors (TCRs) [69]. The peptide antigen presented by dendritic cells to naive T cells may be derived from other *Demodex* antigens or its endosymbiont, *C. kroppenstedtii*. Naive T cells can differentiate into effector T cells if dendritic cells secrete pro-inflammatory cytokines. However, if dendritic cells secrete interleukin-10 (IL-10), naive T cells mature into tolerogenic Tr1 lymphocytes with immunosuppressive functions, and increased *Demodex* density can stimulate IL-10 production via Toll-like receptor 2 (TLR2) expression [68]. 

In the skin lesions of patients with rosacea, we also noticed a statistically significant increase in the abundance of the genus *Bartonella*. *Bartonella* species are Gram-negative aerobic bacteria, facultative intracellular parasites transmitted by vectors. They can infect various cells, including endothelial cells and erythrocytes, leading to long-term diseases such as cat scratch disease, chronic lymphadenopathy, bacillary angiomatosis, vasculitis, and uveitis [70]. Murillo et al. [71] first reported the detection of *Bartonella quintana* 16S rDNA sequences in *Demodex* mites collected from a patient with rosacea. However, since the presence of bacterial DNA does not conclusively prove that *Demodex* could serve as a vector for the transmission of *B. quintana* [72], its vector competence requires additional investigation.

Furthermore, we observed a decreased abundance of the order Peptostreptococcales-Tissierellales and the genera *Anaerococcus* and *Finegoldia* in patients with rosacea which indicates that Gram-positive anaerobic cocci could also potentially play a role in the pathogenesis of rosacea. The results of a previous study [73], which found an enrichment of *Finegoldia magna* in patients with rosacea along with research on *Demodex*, highlight the need for future studies with larger participant groups to accurately identify bacteria and their abundance changes associated with rosacea.

### 4.4. Characteristics of the Skin Microbiota in Patients with Contact Dermatitis

To date, the least amount of knowledge regarding the microbiome has been gathered from patients with contact dermatitis. According to our findings, research on the skin microbiome affected by contact dermatitis in humans has not been published so far. 

Our study showed that the most abundant phylum in the periocular skin lesions in patients with contact dermatitis was Firmicutes. The most abundant genus was *Staphylococcus*, followed by *Corynebacterium*, *Anaerococcus*, *Cutibacterium,* and *Bacteroides.*

LefSe analysis indicated that the potential disease biomarkers could be bacteria of the genus *Paracoccus*, which belong to the phylum Proteobacteria. The abundance of these bacteria was significantly increased in patients with contact dermatitis. Bacteria from the genus *Paracoccus* are part of the healthy microbiome, and some species, such as *Paracoccus yeei*, are associated with opportunistic human infections. The relative abundance of other bacteria was similar in patients with contact dermatitis and healthy individuals, with a slightly higher abundance of Firmicutes and *Staphylococcus* in patients with contact dermatitis. We noticed a decrease in the abundance of phylum Proteobacteria, the class Alphaproteobacteria, and the genus *Cutibacterium* in patients compared to healthy controls. Although the values did not reach statistical significance, these results are important because they are the first to demonstrate the composition of the skin microbiota in patients with contact dermatitis in our study group. Studies on mouse models have indicated that the interaction between *Staphylococcus epidermidis* and keratinocytes may play an important role in the pathogenesis of allergic contact dermatitis, among other factors. *Staphylococcus epidermidis*, a component of the healthy skin microbiota, promotes the production of skin ceramides that contribute to maintaining the skin barrier by secreting sphingomyelinase. Sphingolipids, components of cell membranes, play an important role in cell signaling and immune cell differentiation. They act on keratinocytes through G-protein-coupled receptors called S1P receptors [74]. A study [75] using knockout mice demonstrated that the S1PR2 receptor subtype plays a crucial role in maintaining the skin barrier, but it did not determine which S1PR2 is responsible for this, the one in epidermal keratinocytes or in other cells besides keratinocytes. Conducting future research will contribute to expanding knowledge about the skin microbiome in individuals with contact dermatitis.

### 4.5. Study Limitations

One limitation of this study is the small number of participants in each group. Another potential methodological limitation lies in the classification of disease severity. Also, study controls could only be matched to patients based on age and sex. Since the skin microbiome is dynamic, changing not only depending on age and sex but also by external environmental factors and lifestyle habits (e.g., occupation, climate, use of cosmetics and antibiotics, diet), ideal controls would be healthy individuals with similar lifestyle habits and living in comparable households as patients. Additionally, our study focused on bacteria, which constitute the largest proportion of microorganisms in the skin microbiome. However, the skin also hosts other microorganisms, and their interactions with each other, as well as with the skin, should be considered when analyzing the potential impact of the microbiome on the pathogenesis of inflammatory diseases. 

16S rRNA sequencing typically restricts taxonomic classification to the genus level with its resolution differing across various parts of the phylogenetic tree. Caution should be exercised when differentiating between species using only 16S gene sequencing. Another limitation of 16s rRNA studies to consider is appearance of unknown bacteria which primarily arises from the comprehensiveness of the existing reference databases (in our case, the Silva database). If a bacterial species is not represented in the reference database, it will be classified as “unknown” or may be assigned to a higher taxonomic level (e.g., genus or family). Many microbial species have not yet been cultured, sequenced, or characterized, leading to incomplete databases. Consequently, newly discovered or rare bacteria often cannot be accurately classified. These unknown bacteria may therefore have affected the analysis of the relative abundance of bacteria in our samples. Additionally, 16S rRNA sequencing provide only information on the composition of microbial communities but it does not establish a clear cause-and-effect relationship. It remains to be determined whether skin diseases result from change in the microbiome or cause such a change. Finally, the periocular area is characterized by low microbiome diversity, making it difficult to verify discrete changes even with sophisticated methods such as 16S rRNA sequencing. Further research using whole-genome sequencing is needed to determine the species and strains of bacteria associated with specific diseases. However, despite these limitations, this study has produced statistically relevant data and identified potential bacteria associated with inflammatory skin diseases.

## 5. Conclusions

To our knowledge, this is the first study investigating the microbiota in inflammatory diseases of the periocular region. Our research has brought new information about the composition of the bacterial microbiota in the periocular skin region in both healthy individuals and patients with PD, as well as new insights into bacteria that could play a role in maintaining skin health and potential disease development. *Staphylococcus* and *Corynebacterium* were the most abundant bacterial genera in the microbiota of healthy skin. We assume that the observed changes in the bacterial microbiota on the skin, particularly Gram-positive anaerobic cocci and skin commensals of the genus *Corynebacterium*, could be one of the factors in the pathogenesis of the investigated inflammatory diseases. Understanding these microbial differences may assist in developing new diagnostic tools or targeted therapies that modify the microbiota to restore skin health or prevent disease progression. The identified differences in the microbiota between healthy individuals and patients with periocular dermatitis should be further investigated. 

## Figures and Tables

**Table 1:** Comparison of demographic characteristics between different subgroups of patients with periocular dermatitis and healthy controls.

	Variable	Patients	Controls	*p*-Value
All patients	N	35	39	
	Age, median (range) [years]	58 (22–86)	60 (23–88)	0.455
	Sex, *n* (%)			
	Women	26 (74.29)	23 (59.0)	0.220
	Men	9 (25.71)	16 (41.0)	
Rosacea	*n* (%)	10 (28.57)		
	Age, median (range) [years]	57.5 (36–74)		0.503
	Sex, *n* (%)			
	Women	8 (80.00)		0.288
	Men	2 (20.00)		
Seborrheic dermatitis	*n* (%)	7 (20.00)		
	Age, median (range) [years]	73 (61–82)		0.095
	Sex, *n* (%)			
	Women	4 (57.14)		1.000
	Men	3 (42.86)		
Atopic dermatitis	*n* (%)	12 (34.29)		
	Age, median (range) [years]	37.5 (22–86)		0.023
	Sex, *n* (%)			
	Women	10 (83.33)		0.174
	Men	2 (16.67)		
Contact dermatitis	*n* (%)	6 (17.14)		
	Age, median (range) [years]	61.5 (38–82)		0.841
	Sex, *n* (%)			
	Women	4 (66.67)		1.000
	Men	2 (33.33)		

## Data Availability

The original contributions presented in the study are included in the article, further inquiries can be directed to the corresponding author.
